# LLMs, Truth, and Democracy: An Overview of Risks

**DOI:** 10.1007/s11948-025-00529-0

**Published:** 2025-01-23

**Authors:** Mark Coeckelbergh

**Affiliations:** Department of Philosophy, University of Vienna, Vienna, Austria

**Keywords:** Artificial intelligence, Truth, Democracy, Epistemic agency, Bullshit

## Abstract

While there are many public concerns about the impact of AI on truth and knowledge, especially when it comes to the widespread use of LLMs, there is not much systematic philosophical analysis of these problems and their political implications. This paper aims to assist this effort by providing an overview of some truth-related risks in which LLMs may play a role, including risks concerning hallucination and misinformation, epistemic agency and epistemic bubbles, bullshit and relativism, and epistemic anachronism and epistemic incest, and by offering arguments for why these problems are not only epistemic issues but also raise problems for *democracy* since they undermine its epistemic basis– especially if we assume democracy theories that go beyond minimalist views. I end with a short reflection on what can be done about these political-epistemic risks, pointing to education as one of the sites for change.

## Introduction

Currently there is growing interest in the topic of truth and large language models (LLMs). LLMs are a form of generative AI that can recognize and generate text. They use machine learning (in particular a type of neural network called a transformer model) and are trained on large data sets. LLMs can be used for a wide range of tasks (for example online search and writing code) but perhaps the most famous application is generative AI in the form of chatbots. When given a prompt, chatbots such as ChatGPT (OpenAI), Bard (Google), Llama (Meta), and Bing Chat (Microsoft) produce text in reply. Use of this technology has spread rapidly; it is now widely used across sectors and domains, including politics. For example, political campaigns have used LLMs to interact with voters, answer questions they might have, and send them personalized messages. Microtargeting campaigns use online data to tailor such messages to individuals. LLM technology can also be used to analyse public sentiment on social media regarding political issues, simulate debate scenarios, draft speeches, and create persuasive message to influence politicians and voters.

There are public concerns about truth and LLMs since these technologies do not always give us the truth– sometimes they create fake information– and are used as vehicles of misinformation. As for instance Marcus and Davis (2023) have pointed out, these technologies often produce incorrect or misleading information; they are made to generate responses that *sound* plausible but may in fact be wrong. The systems have been ‘trained to optimize the goal of producing one plausible-sounding word after another rather than actually engage with the meaning of language.’ But LLMs are also used on purpose to spread fake information or to manipulate people. A relevant early example of AI being used for political manipulation purposes is the “Cambridge Analytica” case, in which the 2016 Trump campaign collaborated with a data analysis firm and which involved using data from social media to send tailored messages to specific voter segments in order to influence their political opinions (Cadwalladr & Graham-Harrison, 2018; Rosenberg et al., 2018). Today, LLMs can assist this microtargetting, as they can easily and automatically generate text that resonates with different audiences. There are more knowledge-related concerns. For example, there is the suspicion that LLMs, used in combination with digital social media, may increase political polarisation. In other words, LLMs may be a danger for democracy. Yet in AI ethics and philosophy of AI there is not much academic literature yet that systematically analyses these knowledge-related problems in a way that offers a clear overview and synthesis of the issues and connects them to discussions about democracy.

That being said, in the literature on ethics and philosophy of AI in general there is increasing attention to knowledge-related issues, including issues concerning truth. Consider for instance work on explainability (Wachter et al., 2017), bias (Ntoutsi et al., 2020), epistemic bubbles (Nguyen, 2020), ‘technology-based belief’ (Freiman, 2023), and the influence of AI on what becomes public knowledge (Wihbey, 2024). There is also work on how AI may threaten democracy (Nemitz, 2018; Kreps & Kriner, 2023; Jungherr, 2023; Coeckelbergh, 2023, 2024), which sometimes includes also attention to epistemic issues (see again Wihbey and Coeckelbergh). However, while most of these epistemic problems have been discussed for some years now, a clear overview and synthesis of truth-related problems in the light of LLMs and their implications for democracy is missing. Generally, more work is needed that connects epistemic and political issues raised by AI, including but not limited to LLMs.

This paper aims to assist this project by presenting a brief overview and analysis of how LLMs may create problems for *truth*. I show that and how these are not only epistemological issues but also political ones, in particular problems for *democracy*. I end with a short reflection on what could possibly be done about this, ending with pointing to education as a site for change.

Let me first sketch the political and philosophical context of these truth-related issues.

## Political and Philosophical Context

Truth is a highly contested concept and it is not an exaggeration to say that we are living in and through a crisis of knowledge and a crisis of truth. Truth is fragile, questioned, and attacked. There are not only many philosophical definitions and theories of truth (elsewhere I discuss different truth theories in the light of LLMs), but as for instance a concept such as “post-truth” signals, truth is also a public, indeed a *political* issue. Before LLMs, there were already truth-related problems in contemporary politics. For example, what counts as a reliable source of truth online? What if politicians deceive citizens about the truth or, worse, do no longer care about the truth? How shall democracy deal with pluralism of views about the common good in society? How can we find epistemic common ground, both within nations and globally? What if citizens lack good quality knowledge to participate in decisions? And what is and should be the role of emotions in democracy? How idealistic or realistic should democracy theories be when it comes to assumptions about knowledge? There is no such thing as a Garden of Eden in which there was a perfect democracy and no problems with truth–a situation which is then supposedly destroyed by LLMs. Also before LLMs, democracies already struggled with (1) realizing democracy as an ideal (whichever ideal– there are many) and with (2) the place of knowledge and truth in democracy and in politics.

Indeed, there is a broad question about what the place of knowledge and truth should be in democracy. Traditionally there are two opposing views on this. One is Platonic, epistocratic, and authoritarian. It rejects democracy and sees knowledge and truth as central to the steering of the state (perhaps assuming that truth is objective and that there is only one truth, calling for philosopher-kings). Another is a modern democratic view, which is so “thin” on knowledge and truth that it reduces democracy to a procedure and functional requirements, making no demands at all on knowledge and truth on the part of politicians or citizens, and thus avoiding hard questions concerning truth and democracy. Contemporary political philosophy tends to be situated somewhere between these extremes. Epistemic democracy theories, for instance, move more into the Platonic direction that emphasizes the need for knowledge by aiming for more knowledgeable and more intelligent decisions (Estlund, 2008) and stressing the epistemic benefits of democratic decision making (Landemore, 2013), whereas realistic democracy theories remain closer to the mentioned “thin” ideas, not requiring too much of citizens in terms of knowledge for instance (if anything at all). They adopt Plato’s distrust of citizens’ ability to govern. For example, the minimalist democracy concept is that citizens merely need to express their preferences by voting during elections, choosing between competing elites. Asking more from citizens is seen as unrealistic and (given their lack of knowledge) undesirable. Schumpeter (1942), for instance, argued that democracy is a method of choosing political leaders; the citizens’ role should be restricted to selecting which elites will govern. Deliberative-participative (Habermas, Cohen, and others) and republican theories (Pettit, 1997), by contrast, put more trust in citizens’ abilities to participate in political decision-making but ask that citizens be educated accordingly. They thus present a third way between the horns of the Platonic dilemma (knowledge but no democracy versus democracy but ignorance).

Discussions about LLMs and truth must be situated within this wider political and political-philosophical context, which deserves more analysis on its own. In this paper, which aims to give a brief overview of some key problems regarding LLMs, truth, and democracy, however, I will not further discuss this larger context (or the meanings and definitions of truth, for that matter) but focus on a number of specific phenomena and their significance for, and impact on, democracy.

### Some Truth-related Problems Raised by the Use of LLMs

AI, in the form of LLMs, impacts at least the following truth-related risks and phenomena, which can be briefly enumerated as follows:

1. Misinformation, fake news, and hallucinations (intended or not): The output of an LLM (a particular statement) is not true; it seems that the LLM lies to us.
2. Uncertainty and post-truth: We can no longer distinguish between truth and falsehood; we are uncertain and confused.
3. My/our truth is selected and filtered through polarization and epistemic bubbles: We only (want to) hear the truth from people within our own bubble and it’s difficult for us to revise our beliefs (threat to epistemic agency); we believe that our truth is the only truth or believe that everyone (or every group) has their own truth (relativism).
4. Bullshit and strong relativism: We no longer care about the truth, or at least our machines don’t.
5. Epistemic incest (or epistemic capture or epistemic anachronism): The truth becomes trapped in the past in an AI loop, there is no room for an open future.

Let me discuss each of these issues in more detail:

A first truth problem raised by the behaviour of LLMs is that, when generating text, they do not necessarily produce true statements. It is said that they “hallucinate”. While this metaphor is rather problematic (LLMs are not conscious and hence cannot hallucinate in the sense that humans can do), what is meant is that they unintendedly produce false information. Instead of producing an accurate answer, they make up something, something that has no relation to reality. Generally, the developers of LLMs intend their models to offer an output that is true; they try to avoid hallucinations. But sometimes false information is intended: misinformation and fake news are produced on purpose in order to mislead or manipulate people. For example, advertisers or political campaigners might use LLMs for this purpose. LLMs can be used to spread fake news, damage the reputation of opponents by impersonating them, and target voters with messages that offer misinformation and exploit specific fears and concerns of certain demographic groups. If this is successful, it means that people lack sufficient autonomy and ‘epistemic agency’ to make up their own minds and form their own beliefs. (Coeckelbergh, 2023) Instead, they blindly trust the output of the LLMs, without questioning whether it is true and without forming their own beliefs in a more independent way.

Let me unpack what is going on here. The term ‘epistemic agency’ is used in epistemology to refer to ‘the control that agents may exercise over their beliefs’ (Schlosser, 2019) and relates to the question how our beliefs are formed and revised. The Enlightenment idea is that people want and have control over what they believe: they make up their own minds and can decide to change their beliefs. LLMs may reduce and undermine this control over our belief formation and revision in so far as the technology is used to manipulate the user’s belief formation and prevent their belief revision, or in so far as the technology unintendedly does not encourage the user’s own belief formation and belief revision by presenting its output in a way that appears true. If I am manipulated with misinformation or stuck in LLM’s hallucinations, then, I cannot make up my mind as an autonomous epistemic agent and I will not be encouraged to revise my beliefs– if they are mine at all. Manipulated or mindlessly using the LLM,^1^ I adopt the false belief on the basis of the output given by the LLM, without checking if it is true and without even considering changing it when there is new relevant information. In principle this would be possible (I may become aware of the manipulation or of the limits of the technology), but the technology does not encourage it; instead, it encourages that I go with the output.

While arguably these problems may also occur with for instance Google search or when someone gives wrong advice, LLMs are *made* to produce text that is *convincing*– without including any aim to produce the truth. This renders their (unintended) hallucinations extra problematic. Moreover, due to the scale and speed of related technologies such as digital social media, misinformation can spread rapidly and manipulation can be conducted on a massive scale. And LLMs are also used on purpose to influence voter behaviour, thus becoming part of what Tufekci (2014) has called ‘computational politics’: political campaigns (from the 2016 U.S. presidential election to today) have used so-called microtargeting to deliver personalized political messages to individuals based on their data, thus on purpose manipulating their voter behaviour and spread misinformation.

Second, whereas the first problem still assumes that one can, in principle, relatively easily find the truth and thus contradict the lies, hallucinations, and false statements, in other cases the problem is that it becomes difficult if not impossible to find out the truth, for example because there is too much online misinformation in general (not intended) or because some people on purpose want to create a situation in which it is no longer clear to others what is true and what is not; the uncertainty and confusion is then intended. LLMs might be used for this purpose: one could let hallucinations proliferate to create a climate of epistemic uncertainty and confusion about what is true and false. The term “post-truth” refers to the purposeful creation of such a climate and public sphere. But it might also happen that the climate of uncertainty and confusion is not intended, but still an outcome of the widespread use of the technology.

Third, the previous problems assume that there is something like “the” truth: one truth for everyone. One universal truth, which can be shared. But in so far as people find themselves in a context of polarization and epistemic bubbles (Nguyen, 2020), created by AI, people tend to hear only their own truth: the truth of their own group or bubble. In an epistemic bubble, for example created via social media and AI, people are insufficiently confronted with other beliefs. They are only exposed to beliefs that reinforce their pre-existing beliefs while other beliefs are excluded or ignored. Sometimes they are even actively discouraged from confronting themselves with other views; in so-called echo chambers opposing views are discredited and outside information is deliberately rejected. One’s own view and the view of one’s own group or community is seen as the only truth. Moreover, in so far as AI and other digital technologies lead to a fragmentation of the epistemic landscape, some people might (come to) believe that everyone or every group has their own truth, that there is no universal truth (relativism). Furthermore, because of the bubble effect and the proliferation of relativist views, they might be unwilling and unable to revisit and revise their beliefs, thus not exercising their epistemic agency. Why bother reflecting on, discussing, or revising one’s beliefs if everyone has their own truth or if one is not even exposed to other views? Here the issue is not uncertainty and confusion, but in a sense *too much* certainty: one is completely certain and convinced of one’s own beliefs (and those of one’s group), to such an extent that one no longer listens to others who may have different beliefs and different views.

Fourth, while in the previous cases here is still an interest in the truth– even if only in one’s own truth– one can also imagine a situation in which there is a serious risk that people simply *no longer care* about truth. This can be seen as another form of relativism, but a stronger one than the view that each person has their own truth. Here we have the nihilist idea that truth doesn’t matter anymore at all. In that case, there is no longer an interest in finding out if the output of LLMs is true or not, based on facts or not, etc. People don’t care about it. They believe that it doesn’t matter.

Such a view might be influenced by the fact that LLMs themselves don’t seem to care about truth (or about anything for that matter). As Hicks et al. (2024) have argued, one problem today is that LLMs produce bullshit. Mobilizing Harry Frankfurt’s (2005) understanding of bullshit, the authors argue that LLMs can be said to be bullshitters not just because they produce nonsense or make a factual mistake, but since they are not ‘concerned with truth’ and are ‘designed to produce text that looks truth-apt without any actual concern for truth.’ The problem is then not only that LLMs do not always speak the truth (i.e. produce misinformation) but that they don’t care about truth in the first place. This is true in the sense that the technologies are not designed to do so, they are not designed for truth. Instead, they are rather designed to ‘convey convincing lines of text’– true or not. (3) In other words, they are designed to do what Plato would call producing “rhetoric” instead of telling the truth. LLMs are rhetoric machines. They are sophists. The goal is to convince and influence, not to speak the truth.

Yet since in the case of bullshit there is (usually) no intention to deceive, the authors rightly argue that it would be wrong to see his in terms of lies. Lying is making a false statement with the intention to deceive. Bullshit, then, seems a better term. (4) According to the authors, ChatGPT is ‘a bullshit machine’. (7) While this argument is rather unfair towards rhetoric, which arguably also has value or can make sense, it captures the difference between intended production of misinformation (producing “lies”) and the unintended creation of an epistemic climate in which there is no concern for truth. People might have the impression that both machines and humans are only bullshitting and, ultimately, might themselves not care about the truth anymore.

Finally, even if an LLM is not bullshitting and its output is true, there is the risk that this output starts to refer less and less to the world outside the texts it has been trained on, texts which are from the past. The output might also refer less and less to the world outside the previous outputs. Over time, using LLMs may thus generate a kind of recursivity, an epistemic loop that might well retain truth, but truth that may not be relevant to the world outside and gets stuck in the past. Wihbey (2024) has called this phenomenon of getting stuck in the past ‘epistemic anachronism’ (12) and epistemic ‘capture’ or epistemic ‘lock-in’. One could also call it a kind of *epistemic incest*: a situation in which over time the LLM only interacts with its own input data and output texts. There is no input from new opinions, beliefs, arguments. There is only an output generated on the basis of data from the past (the data on which the LLM has been trained) and data taken from the past output of LLMs themselves. There is truth in the output but the truth gets trapped in the past, so to speak, and becomes less relevant– including less *politically* relevant.

## Why these Issues are Problematic for Democracy

This brings us to the next question. Why are these issues and phenomena problematic for politics? Why worry about them, apart from the obvious corruption of the epistemic landscape and indeed the deterioration of the epistemic capacities and epistemic agency of people? Are there any (other) normative reasons that give them some *political* significance and urgency?

Let me offer some reasons why these epistemic phenomena are problematic for democracy, whatever other reasons there might be to worry about them.

The first issue is a problem for democracy since many democracy theories assume that voters or participants in public debates have some true knowledge and beliefs, for example about the views of political parties and about (problems in) the world, so that they can make up their own mind about who to vote for or what to argue in a public debate. While in practice imperfect knowledge and misinformation may well always have been part of political systems that call themselves democracies (Schumpeter, 1942), several influential *normative* democracy theories prescribe that voters or participants in public debates should form informed decisions and thus would want that voters base their decision on true knowledge. For instance, deliberative and participative democracy theories such Habermas’s (1996) require that citizens deliberate in an open, rational, and informed debate, engaging in public reasoning. This assumes that citizens have access to true knowledge. Similarly, republican democracy theory from Aristotle to Pettit (1997) requires that citizens deliberate about the common good; this also requires access true knowledge, which enables a shared understanding of public interest and the common good. And the more recent epistemic democracy theories (Landemore, 2013; Estlund, 2008) mentioned earlier argue that the quality of democratic decision-making depends on the epistemic quality of voter knowledge (next to adequate procedures and other factors). This again assumes that voters’ decisions should be based on true knowledge. True knowledge is also needed in order to enable people to change their views in a debate, if needed, in the light of knowledge they didn’t have before and knowledge communicated to them by others. Without this epistemic basis for epistemic agency, it is impossible to gain sufficient political democratic agency. Lack of this agency implies that democracy– whether in a thin sense (mere voting) or a thick sense (some form of participation in political debates and decision-making)– does not work (Coeckelbergh, 2023).

The second issue is a problem for democracy since, as Arendt (1951) has observed in *The Origins of Totalitarianism*, confusion about the difference between what is true and false destroys the very possibility of democracy: totalitarian regimes live from an, and actively create, a political-epistemic soil in which the distinction between what is true and what is false is no longer clear. Arendt writes: “The ideal subject of totalitarian rule is not the convinced Nazi or the convinced Communist, but people for whom the distinction between fact and fiction (i.e., the reality of experience) and the distinction between true and false (i.e., the standards of thought) no longer exist.” (Arendt, 1951, p. 474). And even in democracies that are not (yet) totalitarian, there might be the phenomenon of so-called “post-truth politics”: a political condition in which there is public anxiety about what are facts. The condition of anxiety about what is true and what are facts is often stimulated and maintained by right-populist movements, which actively contribute to such a condition and try to benefit from it politically. Again Arendt’s writings are relevant. In *Truth and Politics* (1968) she already warned how factual truth is vulnerable to manipulation by political forces. Alternative versions of reality can undermine democratic discourse. Again, the problem is not just lying as such: when lying is used as a political strategy, the distinction between fact and fiction itself is blurred so citizens can no longer trust anything what is said.

The third issue is problematic since if everyone just sticks to their own political view, is not confronted with other (different) views, and is not willing to revise their (bubble’s or group’s) beliefs at all or even to open them up for discussion, attaining a thicker version of democracy in which citizens participate and deliberate becomes impossible. The latter conception of democracy requires participation in public debate in ways that presumes some openness to listen to others’ views and a willingness to revise one’s own views. But in a fragmented and polarized digital epistemic landscape this becomes difficult if not impossible: everyone stays then in their political trenches. Again epistemic and political agency are impaired, in the sense that there are barriers to the willingness and ability to revise one’s beliefs (Coeckelbergh, 2023). All one can do then is (let people) vote. But the epistemic basis for this is thin. In this situation of epistemic bubbles and fragmentation, there is little sense in convincing people with arguments or in public discussion. There is also little that people share in terms of knowledge and beliefs; they live in different worlds. As a result, there is a lack of mutual understanding and empathy. Debates then become verbally aggressive or even violent. People may insult one another or even threaten one another. They may even (attempt to) eliminate political opponents. Others, maybe because they believe that everyone has their own truth (relativism), may stay away from public fora and social media altogether, given that they are no longer politically safe spaces. Once a minimum of political safety is no longer guaranteed, democratic politics becomes impossible.

The fourth issue, machine bullshitting and not caring about truth at all, is a problem for democracy since in such a climate it is easy for malicious and antidemocratic politicians to manipulate people. If truth becomes a *mere* matter of rhetoric, then the best demagogues and tricksters win, if needed with the help of AI. This supports not only populism but also (keeping in mind Arendt’s argument) authoritarianism, which loves and actively contributes to such a climate, let alone that any “thick” version of democracy is still possible. If there is no concern for a shared or universal truth at all, then it seems impossible to have a real discussion about matters of common concern since there is no agreement possible about what these matters are in the first place. Your political claim is then as good as everyone else’s (see also the previous point) and it is no longer possible to form a basis of shared beliefs or other common epistemic ground needed for participative political discussion. Then discussion on the basis of arguments and facts is no longer possible. Then the public sphere deteriorates into a bullshit space, where nobody is interested in truth (or democracy for that matter) and power is given to those with the best rhetoric. The best bullshitter wins.

Finally, epistemic anachronism, epistemic capture, or epistemic incest are problems for democracy since, as Wihbey (2024) argues, democracy needs new input, needs preferences that might not have been revealed in the past, and needs also knowledge beyond what LLMs might be able to offer, for instance tacit knowledge. Here the problem is not that there is no truth– there might well be truth in the LLM’s output– but that this is a truth from the past, an irrelevant by-product of AI, not a truth we need today in and for our democracies. If democracies are supposed to pick up new signals from citizens, but if only past signals are reiterated, the knowledge available to democracy is no longer up to date and no longer relevant. Then political activities such as ‘agenda-setting, framing of narratives, and selection of information’ are outdated since ‘based on an epistemically anachronistic model.’ (24) The thick ideal of democracy, which includes citizen participation and deliberation, is equally in trouble since it needs updated information and knowledge that is relevant for now. Why argue and deliberate on the basis of outdated knowledge?

Moreover, humans might have types of knowledge about the current situation that do not make it into the input for the LLMs but that may nevertheless be politically relevant, for example intuitions, emotions and/or (other kind of) embodied kind of knowledge. Wihbey warns that AI in general may not be very good in picking up signals related to tacit knowledge: if ‘’what is considered true, interesting, and useful by humans is continually mediated by AI, which then reinforces past ideas and preferences,’ then ‘humans may not be able to access emerging, organic ground-truth signals from fellow citizens about their dispositions on issues, potentially silencing important intuitions, emotions, tacit knowledge, gut feelings, and experiences.’ (13) This narrowing down of truth to past truths and truths in the form of dispositions that can be easily be made explicit and put into text, is not good, if not disastrous for democracy, which is supposed to take into account, and deliberate about, what is true and interesting now and what citizens think and feel now. And the latter kind of knowledge may be at least partly situated outside the texts that circulate in the incestuous LLM data economy. Humans may pick up such signals, intuitions, and emotions; machines lack this embodied and situational kind of knowledge. This means that while they may pick up on some texts in which these politically relevant emotions and intuitions are expressed, they are likely to miss the relevant texts and non-textual expressions because they lack that kind of knowledge, awareness, and subjectivity.

This is also problematic if, as Mouffe (2005) has argued, emotions are essential to an engaged and participative democratic politics, which is not only about reasoned debate but also about political engagement and struggle motivated by emotions and passions. Coming from another political direction, Nussbaum (2013) has argued that emotions play a vital role in motivating individuals to care about others and engage in the political process. While emotions in politics can also be problematic, if emotions are excluded from political knowledge altogether via LLMs, then this is a threat to democracy.

The narrowing down of truth and knowledge to past truths and truth in the form of explicit knowledge is also problematic since with its output LLMs may make it seem as if everything is already known, as if the truth is known and knowledge is complete. Innerarity (2024) has suggested that whereas democracy always involves decision-making under incomplete knowledge and uncertainty, AI falsely gives us the illusion of epistemic certainty and completeness, leaving out the ambiguities and knowledge gaps that are inherent in the political life. This is not only an argument against AI taking over politics (Innerarity’s point, and something Arendt may be interpreted as having warned against, see Arendt, 1972); it is also a problem for using AI, in the form of LLMs, in democracies in general. Acknowledging uncertainty, incompleteness, and indeed limits to one’s own knowledge seem essential for participating in, and creating, a healthy democracy.

## What to do about These Political-Epistemic Risks?

Here are a number of ways in which these technology-pushed epistemic risks, in this paper conceptualized as problems regarding truth, could be mitigated. Here my aim is not to present original views, arguments, or analysis, but simply to offer some avenues for dealing with the risks– most of which are well-known when it comes to governance of AI in general, but some of which require more attention (the role of traditional media and education):

1. Research on, and policy development in the area of, ethical-epistemic rules for LLMs and on how to operationalize them in practice.
2. Regulation and oversight of the tech industry in ways that influence and shape the development of new LLM technology (and more generally new AI tech) according to the mentioned ethical-epistemic rules.
3. Quality (human) journalism that also protect these rules.
4. Education that raises awareness of these problems and, more generally, of the epistemic limitations and political risks of LLMs (and other types of AI).

First, currently there are plenty of lists of ethical principles that can guide AI policy, but so far, less attention has been paid to the epistemic risks involved in using AI, including LLMs. More investment in research projects, preferably interdisciplinary ones, that help to clarify and understand these risks and work out ways to mitigate the problems is badly and urgently needed, especially in the light of the mentioned risks for democracy. In terms of policy, we need rules that can work at the operational level in policy and innovation, not only in the form of ethical norms but also technical standards, labels, and incentives.

Second, these policies should inform effective regulation. LLM technology should not be taken as a natural phenomenon; the mentioned risks are risks of the current systems, and as humanity and as societies we have the possibility to shape their future development. Regulation and oversight should make sure that the technology is developed in such a way that democracy is supported instead of undermined (Coeckelbergh, 2024). This should include nurturing and taking care of its epistemic basis, and LLMs should be evaluated and regulated in this light. Currently mitigating the epistemic-democratic risks created by LLMs is left too much in the hands of Big Tech.

Third, digital social media, in which some of the risks discussed in this paper can become imminent, are not the only media we have; we also still have the traditional media such as newspapers and TV. Traditional quality media and independent journalism can and should play an important role to protect democracy from threats due to new technologies, and actively help to promote some of the “thick” normative democratic values that underpin this analysis: participation, deliberation, and engagement. For example, instead of providing more fuel for heated conflict and polarization (which often happens because owners of these media want more audience in order to make more money), these media and journalists could in principle try to spark real political discussion, that is, not just facilitating the expression of entrenched and dogmatic views but a real debate in which there is also an effort to find some common ground, both in terms of shared knowledge, truth, and beliefs and in terms of a vision of the future. This may also help to relieve the broader knowledge crisis mentioned in the beginning of this paper. But it requires re-thinking regulation and potentially ownership of media.

Finally, users of LLMs and citizens should also not be taken as a kind of fixed factor. It may be, as it is often said, that people get the politicians they deserve, and perhaps it is also the case that people get the technologies they deserve. But education can change people. Currently education tends to be shaped by minimalist views of democracy, if the topic is dealt with at all. But this situation can be changed. If we improve political education in a way that includes civic education that supports richer democracy ideals (more participative and republic ideals, which require a stronger epistemic basis) and make people aware of the epistemic and political risks of LLMs and other AI technology, citizens can make better choices, have better debates about technology, and ultimately themselves *contribute* to the creation of better politics and better technology. That’s, at least in part, what democracy should be about.

## Footnotes

1 Note that manipulation is not the same as mindless use. In the case of manipulation, a manipulator tries to influence the one who is manipulated in a way that is in line with the manipulator’s aims. Usually this is understood as involving hidden influence or deception, although this criterion has been questioned (Klenk, 2022. In the case of mindless use, however, there is no manipulator or manipulation at work. The effects on one’s epistemic agency are not intended.
